# Psychological Distress, Depression, and Anxiety in Nursing Students: A Longitudinal Study

**DOI:** 10.3390/healthcare11050636

**Published:** 2023-02-21

**Authors:** Yonca Sonmez, Meltem Akdemir, Ayse Meydanlioglu, Mehmet Rifki Aktekin

**Affiliations:** 1 Department of Public Health, Akdeniz University Faculty of Medicine, Antalya 07058, Turkey; 2 Department of Public Health Nursing, Akdeniz University Faculty of Nursing, Antalya 07058, Turkey

**Keywords:** psychological distress, depressive symptoms, anxiety, perceived stress, nursing students, longitudinal study

## Abstract

The aim of this longitudinal study is to determine how stressful life events, psychological distress, depressive symptoms, and anxiety change in a cohort of students from one nursing faculty during the education process and to document the factors related to psychological distress, depressive symptoms, and anxiety during the fourth year of education. The General Health Questionnaire (GHQ-12), the Beck Depression Inventory (BDI), and the State-Trait Anxiety Inventory (STAI) were applied to students within the faculty of nursing within the first week of the 2018–2019 academic year. All students were asked to answer a questionnaire measuring their possible stressful life events (first timepoint). The process was repeated for the same students again in the fourth year (second timepoint). The changes between the two timepoints were examined. Nursing students’ GHQ-12 and STAI scores and averages increased significantly from first timepoint to second timepoint (*p* < 0.05). There was a significant increase in the prevalence of depressive symptoms for the ≥21 cut-off point of BDI in the fourth year of the study cohort. A significant increase in perceived stress levels between the two timepoints was also found for numerous stressful life events. As a result of linear regression, “dissatisfaction with major” was found as a determinant on all scale scores. The psychological indicators increased significantly in nursing students during their education. Interventions to reduce stress, anxiety, and psychological distress are needed to improve the mental health status of nursing students.

## 1. Introduction

Poor mental health among university students is a worldwide concern. Depression and anxiety symptoms are commonly reported among university students in many regions of the world and they impact the life quality and academic attainment of students [1]. Many studies in the literature have reported that university students have a higher risk of mental health problems compared to the general population of their peers. University students are a vulnerable group due to the developmental stage they are at in life, the nature of adaptation to the new environment at university, the academic or parental expectations, and the intensity of higher education programs [2,3,4,5].

According to two different meta-analysis studies, the risk factors for depression and anxiety in university students were sex (female), academic stress/academic difficulty, smoking, traumatic experiences, poor sleep quality, poor family economic status, no self-confidence/low self-esteem, irregular eating habits, internet addiction, drinking, academic grade (non-freshmen), family origin (rural), low educational level of father/mother (≤9 years), and negative life events in childhood [3,6].

In addition to the difficulties experienced by other students, nursing students have to cope with some other issues specific to their training in the field of health [7]. In this context, there are studies in the literature showing that stress, depression, and anxiety are quite common in nursing students in different countries of the world [8,9]. In a meta-analysis of 27 cross-sectional studies conducted by Tung et al. in 2018, the prevalence of global depression in nursing students was reported as 34.0%. This suggests a high prevalence of depression among nursing students in comparison to the general population, which was 4.7% [10].

Multiple factors that contribute to stress and impact the mental health of nursing students have been identified in the literature. These stressors can be categorized as either academic or clinical in nature. Academic stressors include exams, research projects, class assignments, poor grades, and strained relationships with faculty members. Clinical stressors, on the other hand, include adjustments to new clinical environments, inadequate nursing knowledge and skills, fears of making mistakes, conflicts with peers and senior nurses, concerns about patient care, including terminal patients, weak connections with patients and their families, excessive workload, and witnessing traumatic events such as death [11,12,13].

It is crucial to assess the mental health of nursing students and implement mental health interventions to support this vulnerable group. The stress, depression, and anxiety experienced by nursing students can have negative impacts on their own health, and also affect their academic performance and the quality of care they provide to patients. Nursing is a profession that requires empathetic human interaction. Depression and anxiety can impair cognitive processes and communication skills, leading to poor clinical decision making and increase the risk of medical errors in practice. Maintaining the mental well-being of future healthcare providers is a crucial aspect of nursing education. Additionally, the psychological well-being of employees has a direct impact on employee turnover and job performance, making it a critical concern for the human resources management of the nursing workforce [14,15,16].

Although there are cross-sectional studies investigating the levels of stress, prevalence of psychological distress, depressive symptoms, anxiety, and related factors in nursing students, there is limited data evaluating the change over time. No longitudinal study evaluating the stress levels and mental health of nursing students in Turkey has been found in the literature. This study is a first in Turkey in that respect. The aim of the study is to determine the change of stressful life events, psychological distress, depressive symptoms, and anxiety in a cohort of students from one nursing faculty throughout certain timepoints in their education process and also to document the factors related to psychological distress, depressive symptoms and anxiety during the fourth year of education.

## 2. Materials and Methods

### 2.1. Study Design

This research was a longitudinal prospective study aiming to monitor the students at a nursing faculty in terms of stress and psychological indicators (psychological distress, depressive symptoms, and anxiety) throughout certain timepoints in their educational process.

### 2.2. Study Setting, Participants, and Data Collection

The research cohort consisted of all students newly enrolled in Akdeniz University Faculty of Nursing in the 2018–2019 academic year. Detailed and self-reported questionnaires were applied to all newly enrolled nursing students in 2018 within the first week of the first semester (first timepoint). The second questionnaire form, which included the same information, was applied to the same students at the end of the fourth year (second timepoint). Questionnaires were scheduled so that the students did not have any exam two weeks before and after the application.

In Turkey, a four-year program for a bachelor’s degree in nursing is granted to students who have completed a total of 240 European Credits Transfer System. Courses in the Faculty of Nursing are carried out according to the classical education model. In the first year, basic medical science courses are included in the curriculum and starting from the second year, clinical applications are integrated into theoretical courses. In the fourth year, students mainly practice in the hospital. Students take courses in psychology and communication such as Interpersonal Relations, Psychology, Developing Communication Skills, Professional Communication, Mental Health, and Disease Nursing.

The number of students who initially enrolled and were accepted to participate in the research in the first year was 202. As 15 students dropped out of the faculty and 33 students did not want to answer the questionnaire in the fourth year, the rate of follow-up was 76.2% (154/202).

### 2.3. Measures

The data for the study were collected using a Personal Information Form (PIF), Stressful Life Events Form (SLEF), the General Health Questionnaire-12 (GHQ-12), the Beck Depression Inventory (BDI), the State-Trait Anxiety Inventory (STAI), and the Internet Addiction Test (IAT).

The PIF includes questions about age, sex, family structure, residence for the longest period, educational status of parents, economic status of the family, perceived health status, living arrangement, sleeping duration, presence of chronic disease, regular exercise habits, and smoking/alcohol use.

The SLEF consists of 23 items that can cause stress. Each item was scored between 0 (not at all stressful) and 10 (always/very stressful) according to the level of stress perceived by the students. The mean score of each item was evaluated separately for two timepoints.

The GHQ-12 was used to measure current psychological distress [17]. The Turkish validity and reliability study of the scale was performed by Kılıç [18]. The form has 12 questions, each of which has a 4-point response scale, which are scored from 0 (least distress) to 3 (most distress). The total score of the GHQ-12 can be calculated by summing the 12 items. The minimum score for the GHQ-12 is 0 and the maximum score is 36. Higher scores indicate greater levels of psychological distress. The cut-off value was taken as ≥12 to determine the prevalence of psychological distress as suggested by Goldberg et al. [19]. The internal consistency of the GHQ-12 measured in our study using Cronbach’s alpha was 0.86.

The BDI was developed by Beck et al. [20] and adapted for use in Turkey by Hisli [21]. Consisting of 21 items, the BDI is used to assess depressive symptoms on the basis of a 4-point Likert-type scale. Possible scores on the BDI range from 0 to 63, with higher scores indicating higher levels of depression. Although the authors who developed the Turkish version of the BDI have suggested ≥17 as the cut-off value for the prevalence of depressive symptoms, different cut-off points (≥10, ≥15 and ≥21) are used in the literature [10]. Therefore, the prevalence of depressive symptoms was calculated for different cut-off values. In this study, Cronbach’s alpha of the scale was 0.89.

STAI is a 40-item, self-report questionnaire with two subscales: a 20-item form for State Anxiety (S-Anxiety) and a 20-item for Trait Anxiety (T-Anxiety) [22]. S-Anxiety can be defined as a transient momentary emotional status resulting from situational stress. T-Anxiety refers to a predisposition to react with anxiety in stressful situations. All items are rated on a 4-point scale (from “Almost Never” to “Almost Always”). Each form allows a minimum score of 20 and a maximum score of 80. Higher scores indicate greater anxiety. A cut point of 39/40 was used to detect clinically significant symptoms for S-Anxiety. A valid cut-off score is not preferred for T-Anxiety scores [23]. A valid and reliable Turkish version of STAI was applied in this study [24], while the Cronbach’s alpha value for S-Anxiety was 0.87 and 0.86 for T-Anxiety.

IAT was developed by Young and comprises a 20-item questionnaire to be answered using a Likert scale [never (0) to always (5)]. A composite score for IAT can range from 0 to 100 where a greater score is indicative of greater internet addiction [25]. Turkish validity and reliability of the scale was adapted by Bayraktar [26]. Total scores that range from 0 to 30 points are considered to reflect a normal level of internet usage. Scores of 31 to 49 indicate the presence of a mild level of internet addiction; 50 to 79 reflect the presence of a moderate level; and scores of 80 to 100 indicate a severe internet addiction. In this study, ≥50 was taken as the cut-off value for the prevalence of internet addiction.

### 2.4. Ethical Conciderations

Written approval to conduct this study was obtained from the Clinical Research Ethics Committee of Akdeniz University (date/number: 2018/712). Verbal and written consent to participate in the study was received from all participants. The study was conducted according to the ethics guidelines set out in the Declaration of Helsinki.

### 2.5. Data Analysis

Study data were evaluated using SPSS (Statistical Package for the Social Sciences) version 23.0 (IBM, Armonk, NY, USA). Descriptive statistics were defined by number, percentage, mean, standard deviation, and minimum-maximum values. The paired samples t-test was used to compare means, and McNemar’s test was used to compare frequencies in the paired groups. Reliability analyses of the scales were performed using Cronbach’s alpha coefficients. Pearson’s correlation coefficient test was used to evaluate the relationship between GHQ-12, BDI, and STAI. Multiple regression analysis was used to define predictor factors for psychological distress, depression, and anxiety scores. For this, four separate multiple regression models (backward elimination method) were created. GHQ-12, BDI, S-Anxiety, and T-Anxiety scores (second timepoint) were determined as dependent variables. Nine stressful life events with the second timepoint score above 4 (financial problems, worrying about the future-individual, worrying about the future-communal, high expectations of the family, worrying about exam, accommodation problems, family-oriented problems, dissatisfaction with major, and dissatisfaction with social activities) and other variables (sex, age, doing regular physical exercise, smoking, perceived health status, and IAT score) were added to the model as independent variables. Sex and age were added to the model because they were found to be significant in the literature and could also be confounders. Since there was a significant relationship between doing regular physical exercise, smoking, perceived health status, IAT score, and psychological scale scores in a binary analysis, these variables were included in the regression model. The level of significance for statistical tests was established as *p* < 0.05.

## 3. Results

The age and sex distributions of the students at the first timepoint and the second timepoint are shown in Table 1. Of the research cohort of 202 nursing students, 81.2% lived in a type of nuclear family, while 43.1% lived in a district for the longest time. The rate of students whose mothers had nine years or more education was 15.9%, while this rate was 26.7% for their fathers. In total, 28.2% of the students were doing regular physical exercise, 16.8% were smokers, and 21.8% perceived their health as fair/poor.

According to the results of the analysis, it was observed that the scores of all scales, except BDI and some perceived stressful life events’ scores, increased significantly between the two timepoints (Table 2).

The life events that cause the most stress and increase the level of stress significantly from the first to the fourth year were “financial problems” and “worrying about the future (individual and communal)”. On the other hand, “alienation from the city” decreased significantly during the four years in nursing students (Table 2).

### 3.1. General Health Questionnaire-12 Total Scores and Psychological Distress Prevalance

Both the GHQ-12 total score (Table 2) and the prevalence of psychological distress (Table 3) of the nursing students increased significantly from the first to the second timepoint (*p* values 0.001 and 0.002, respectively).

### 3.2. Beck Depression Inventory Scores and Depressive Symptom Prevalance

Although the BDI total score showed a slight increase from the first timepoint to the second timepoint, it was not statistically significant (Table 2).

When comparing the prevalence of depressive symptoms for the different cut-off points, there was an increase for all in the fourth year, but a significant increase was found only for ≥21 cut-off points (8.4%, 16.2%, respectively; *p* = 0.043). The results are presented in Table 3.

### 3.3. State-Trait Anxiety Inventory Scores and State-Anxiety Prevalance

The S-Anxiety and T-Anxiety scores of the nursing students increased significantly in the second timepoint (*p <* 0.001 and *p* = 0.006, respectively). The results are presented in Table 2.

According to the 39/40 cut-off point, the prevalence of S-Anxiety was 45.5% in the first timepoint and 63.6% in the second timepoint. The increase in prevalence of S-Anxiety between the two timepoints was statistically significant (*p* = 0.001) (Table 3).

### 3.4. Correlations

Pearson correlation coefficients among GHQ-12, BDI, and STAI scales for the second timepoint are shown in Table 4. Both the S-Anxiety and T-Anxiety scores showed a moderately positive and significant correlation with GHQ-12 and BDI scores (*p* < 0.001 for all).

### 3.5. Multiple Regression Analysis

As a result of multiple regression analysis, “dissatisfaction with major” was found to be the predictor variable on all psychological scale scores.

On the other hand, “dissatisfaction with social activities” was found to be a risk factor for all scales except T-Anxiety, and a high IAT score was found to be a risk factor for all scales except GHQ-12.

Not doing regular physical exercise and “worrying about the future (individual)” were decisive on the GHQ-12, whereas “worrying about exam” was decisive with the BDI score. Fair/poor perceived health status was associated with the S-Anxiety score, and finally, “worrying about the future (individual)” was associated with the T-Anxiety score. The results are presented in Table 5.

## 4. Discussion

We set out to investigate psychological distress, depressive symptoms, and anxiety in student nurses using validated instruments within a longitudinal cohort design. At the same time, we examined the predictors of mental health in nursing students who came to the end of their educational process.

According to the results of the study, the GHQ-12 and STAI scores of the students increased significantly at the end of their education, and when the prevalence was evaluated according to the cut-off points, the presence of psychological distress and state anxiety was found in more than half of them. There was a significant increase in the prevalence of depressive symptoms only for the ≥21 cut-off point of BDI at the end of the education, but no significant change was found at the lower cut-off points. The collection of second timepoint data after the COVID-19 pandemic may have caused students to experience mental, economic, and parental problems and increased anxiety. Similar results were obtained in the follow-up study conducted with students at the faculty of medicine of the same university before the pandemic. It was determined that psychological indicators deteriorated in the first two years of education in medical students [27], even without the conditions of a global pandemic. This is important in terms of showing the necessity of monitoring mental health and taking precautions in the education process of future health professionals. Detection and treatment of students with moderate to severe depression is vital to prevent possible suicides and dropouts. There are few longitudinal studies conducted in different countries in accordance with our results showing that emotional problems increase during nursing education [28,29,30].

Our findings show that many stressful life events, apart from the psychological scales, increase significantly at the end of nursing education. We found that senior students experience both academic and clinical stressors with higher intensity. Our results are similar to the literature [11,31]. Senior students may feel more anxiety about the future and find themselves on the verge of taking on professional responsibility. This can lead to a deterioration in their mental health. “Worrying about the future–individual” as a risk factor for psychological distress and T-Anxiety as a result of the regression analysis of this study also support this interpretation. In Turkey, nursing students take an exam to work in public institutions and organizations after graduation, and are then assigned to a city in the country according to their scores. Students with low scores in the exam either cannot work or have to look for a job in private health institutions. If individual future anxiety cannot be dealt with, it may cause the deterioration of students’ mental health.

According to the results of the regression analysis of this study, some stressful life events other than “worrying about the future–individual” were also found to be an important predictor of mental health. Among them, “dissatisfaction with major” is remarkable. There are studies in the literature showing that the psychological indicators of nursing students who voluntarily choose the profession are better [7,32,33]. Many positive outcomes occur when a young individual knows the working conditions of the profession they are training for and studies with intent. Therefore, it can be said that conscious career choice can be an important variable on the mental health of individuals. From this point of view, in order to prevent dissatisfaction with the choice of profession, it can be suggested that nurses and academicians they work with during internships should be positive role models, and students should be included in activities to gain a positive perspective on the profession throughout the education process, and universities and hospitals should encourage practices that will emphasize the importance of the nursing profession not only to sick individuals but also to healthy individuals [34,35].

Another risk factor for psychological indicators was determined as “dissatisfaction with social activities”. Similar results were found in other studies as well [27,36]. This situation shows that the students studying in the departments related to human health cannot allocate enough time for themselves due to the intensity of education, thus increasing their distress and stress. For this reason, the inevitably intense nursing education should be planned in a way that will enable social activities, encourage participation, and protect students’ mental health.

In the literature, there are many studies that indicate a positive relationship between negative life events and stress levels and psychological morbidity, and a negative relationship with psychological well-being [7,12,30,36,37,38]. These results demonstrate the importance of focusing on reducing students’ stress levels. Stress is also recognized by the World Health Organization as one of the top ten determinants of health inequalities, and it has been accepted as a very important indicator for the early diagnosis of students at risk for mental health problems on university campuses [4].

According to the results of the multivariate analysis, another finding was that the GHQ-12 scores of the students who did not exercise regularly were higher. Similar to our results, there are studies in the literature showing that regular physical exercise is positively associated with mental health [36,39]. This shows us that physical facilities and curricula that encourage students to exercise should be provided.

The results of the study showed that the internet addiction score increased significantly in the fourth year and high scores were a risk factor for both depressive symptoms and anxiety. Many studies have been found in the literature on the association of internet addiction with depressive symptoms and other psychosomatic symptoms [36,40,41]. The younger generation is more vulnerable to internet addiction, whether they use it for entertainment, communication, or academic purposes. The pandemic and the disconnection from individual face-to-face communication that we all experience have pushed students toward the virtual world even more. This may have caused young people to lose some of their social communications in physical, everyday life, which is a very serious trigger for the development of depressive symptoms in the long term. It is important to direct students to face-to-face social activities from the first grade and to be in touch with them in terms of internet addiction. It is necessary to raise awareness of the negative effects of internet addiction in students and to encourage them to use the internet efficiently.

In addition, positive and significant correlations were found between the scores of the GHQ-12, BDI, and STAI in this study, and this finding is consistent with the literature [15,32,42].

According to the literature, stress, anxiety, and depression in undergraduate students are directly attributed to their learning environments, student diversity, and course content [14]. Therefore, university administrators should provide an educational environment that minimizes the risk of stress, anxiety or depressive symptoms in nursing students. According to a systematic review that included 22 studies, different effective interventions have been reported to reduce stress, anxiety, or depression among nursing students. Studies of mindfulness-based interventions comprised the largest sample sizes and displayed the highest levels of evidence [14]. In addition, it has been shown that mindfulness training, when combined with physical education, provides significant improvements in reducing depression among nursing students [43].

Some limitations of the study should be recognized. Firstly, the fact that the research was conducted in a single university makes it difficult to generalize the results. Secondly, our study relied on self-report measures. Due to the use of self-administered questionnaire, the study might have suffered from social desirability bias. Furthermore, some of the students could not be reached at the second follow-up due to drop-out and refusal. Students who cannot be reached may have dropped out of school due to poor psychological indicators or may have chosen not to participate in the research. Therefore, the results obtained should be interpreted with caution.

## 5. Conclusions

The deterioration in psychological indicators during the nursing education process showed that there is a need for interventions to reduce stress, anxiety, and depressive symptoms in nursing students. It would be appropriate to provide one-on-one counseling services by faculty members and to equip students with effective stress coping methods. At the same time, peer mentoring by the upper classes can also contribute positively to the process by experience exchange.

In order to eliminate negative thoughts about the nursing profession in the education process, a more collaborative and supportive environment should be created with the teaching staff and clinical personnel. In this study, we examined the individual risk factors affecting the mental health of nursing students. However, some structural problems such as employment, working conditions, and wage policies should not be ignored in the nursing profession in Turkey.

Lastly, there is a need for more multicenter, large-scale longitudinal studies on this subject. Research supported by the qualitative dimension can provide valuable information about stressors and their psychological problems, causes, and coping methods during nursing education.

## Figures and Tables

**Table 1:** Descriptive characteristics of nursing students at the first and second timepoints.

Demographic Characteristics	First Timepoint	Second Timepoint
Participants (n)	202	154
Age [Mean ± SD (Range)]	18.6 ± 1.2 (17–29)	22.3 ± 0.9 (21–27)
Sex		
Female [n (%)]	140 (69.3)	113 (73.4)
Male [n (%)]	62 (30.7)	41 (26.6)

Abbreviations: SD, Standard Deviation.

**Table 2:** The scores of nursing students from the scales in the first and second timepoints and their perception of stressful life events (*n* = 154).

	First Timepoint	Second Timepoint	SD *	*p* ^†^
	Mean ± SD	Mean ± SD		
Instruments (min–max scores of scales)				
GHQ-12 (0–36)	11.1 ± 5.6	12.7 ± 5.6	6.30	0.001
BDI (0–63)	8.5 ± 7.7	9.2 ± 9.8	9.97	0.384
S-Anxiety (20–80)	39.2 ± 9.1	42.6 ± 9.4	10.84	<0.001
T-Anxiety (20–80)	43.3 ± 8.7	45.3 ± 8.5	9.13	0.006
IAT (0–100)	25.3 ± 13.1	32.1 ± 17.2	19.8	<0.001
Stressful life events (0–10)				
Financial problems	4.9 ± 3.0	6.9 ± 2.6	3.36	<0.001
Family-oriented problems	3.2 ± 2.7	4.6 ± 2.6	3.19	<0.001
High expectations of the family	4.7 ± 3.3	5.2 ± 2.8	3.64	0.122
Living away from home	3.2 ± 3.0	3.8 ± 2.8	3.69	0.076
Alineation from the city	3.1 ± 2.8	2.4 ± 2.3	3.37	0.013
Dissatisfaction with social activities	3.2 ± 2.7	4.2 ± 2.9	3.35	0.001
Worrying about the future–individual	4.7 ± 3.1	6.9 ± 2.8	3.33	<0.001
Worrying about the future–communal	4.8 ± 3.2	6.8 ± 3.1	3.63	<0.001
Accomodation problems	3.0 ± 2.9	4.7 ± 3.1	3.78	<0.001
Dissatisfaction with major	3.1 ± 3.0	4.3 ± 2.8	3.22	<0.001
Adaptation to the university environment	2.4 ± 2.4	2.2 ± 2.4	3.19	0.351
Risk of educational failure	2.0 ± 2.4	3.8 ± 2.9	3.56	<0.001
Worrying about exam	4.7 ± 2.8	5.0 ± 2.9	3.68	0.432
Difficulty in making friends	2.3 ± 2.2	2.7 ± 2.5	2.78	0.069
Romantic relations	2.4 ± 2.6	2.8 ± 2.6	3.34	0.149
Problems with physical appearance	2.5 ± 2.8	3.2 ± 2.7	3.11	0.002
Political and moral pressure	1.3 ± 2.2	3.3 ± 3.1	3.27	<0.001
Alcohol use	0.9 ± 2.6	1.4 ± 2.4	3.13	0.033
Drug use	0.9 ± 2.7	1.0 ± 2.3	3.30	0.787
Mental discomfort	1.3 ± 2.6	2.0 ± 2.8	3.26	0.007
Physical illness	1.3 ± 2.5	2.2 ± 2.9	3.18	<0.001
Exposure to physical violence	0.9 ± 2.6	1.4 ± 2.6	3.20	0.063
Exposure to psychological violence	1.4 ± 2.7	2.6 ± 3.1	3.51	<0.001

Abbreviations: GHQ-12, General Health Questionnaire; BDI, Beck Depression Inventory; S-Anxiety, The State Anxiety Scale; T-Anxiety, The Trait Anxiety Scale; IAT, Internet Addiction Test. * Standard Deviation of the paired differences, ^†^ Paired t test.

**Table 3:** Psychological morbidity prevalence of nursing students in the first and second timepoints (*n* = 154).

Scales and Cut-Off Points	First Timepoint		Second Timepoint		
	n	%	n	%	*p* *
Psychological distress					
GHQ-12 ≥ 12	65	42.2	87	56.5	0.002
Depressive symptoms					
BDI ≥ 17	24	15.6	30	19.5	0.392
BDI ≥ 21	13	8.4	25	16.2	0.043
State anxiety					
S-Anxiety ≥ 40	70	45.5	98	63.6	0.001
Internet addiction					
IAT ≥ 50	8	5.2	25	16.2	0.002

Abbreviations: GHQ-12, General Health Questionnaire; BDI, Beck Depression Inventory; S-Anxiety, The State Anxiety Scale; IAT, Internet Addiction Test. * McNemar’s Test.

**Table 4:** Correlation coefficients among GHQ-12, BDI, and STAI.

Psychological Scales	GHQ-12	BDI	S-Anxiety
GHQ-12	1.000		
BDI	0.551 *	1.000	
S-Anxiety	0.509 *	0.612 *	1.000
T-Anxiety	0.473 *	0.512 *	0.655 *

Abbreviations: GHQ-12, General Health Questionnaire; BDI, Beck Depression Inventory; S-Anxiety, The State Anxiety Scale; T-Anxiety, The Trait Anxiety Scale. * *p* < 0.001.

**Table 5:** Results of multiple regression analyses for nursing students.

Psychological Scales	Independent Variables in the Model (Constant)	R^2^	B	SE	Beta	t	*p*
GHQ-12 (0–36)	Dissatisfaction with social activities	0.366	0.641	0.146	0.333	4.376	<0.001
	Worrying about the future—individual		0.342	0.148	0.170	2.307	0.022
	Dissatisfaction with major		0.351	0.146	0.175	2.405	0.017
	Not doing regular physical exercise		1.770	0.787	0.151	2.250	0.026
	Smoking		1.590	0.824	0.130	1.928	0.056
BDI (0–63)	Dissatisfaction with social activities	0.352	0.543	0.257	0.160	2.118	0.036
	Dissatisfaction with major		0.720	0.281	0.204	2.560	0.011
	Worrying about exam		0.496	0.240	0.148	2.067	0.040
	IAT		0.163	0.042	0.287	3.843	<0.001
S-Anxiety (20–80)	Dissatisfaction with social activities	0.385	0.693	0.238	0.214	2.917	0.004
	Dissatisfaction with major		0.768	0.257	0.228	2.993	0.003
	IAT		1.176	0.039	0.323	4.547	<0.001
	Fair/poor perceived health status		3.053	1.532	0.135	1.993	0.048
T-Anxiety (20–80)	Dissatisfaction with major	0.364	0.635	0.238	0.209	2.665	0.009
	Worrying about the future—individual		0.465	0.218	0.153	2.134	0.035
	IAT		0.169	0.036	0.344	4.693	<0.001
	High expectations of the family		0.384	0.225	0.124	1.705	0.090

Abbreviations: GHQ-12, General Health Questionnaire; BDI, Beck Depression Inventory; S-Anxiety, The State Anxiety Scale; T-Anxiety, The Trait Anxiety Scale; IAT, Internet Addiction Test; R^2^, R Squared; SE, Standard Error.

## Data Availability

Data is available upon request from the corresponding author. Data are not publicly available due to privacy and ethical constraints.
